# Early Clinical and Echocardiographic Outcomes of Tricuspid Transcatheter Edge-to-Edge Repair in Asian Patients

**DOI:** 10.1016/j.jacasi.2025.10.004

**Published:** 2026-01-06

**Authors:** Kwong-Yue Eric Chan, Ho-On Alston Conrad Chiu, Simon Cheung-Chi Lam, Martin Kin-Lam Cheng, Pik-Ki Law, Ka-Chun Un, Cheuk-Wing Jonathan Lee, Shu-Yue Sze, Yui-Ming Lam, Frankie Chor-Cheung Tam, Daniel Tai-Leung Chan, Gilbert H.L. Tang, Chun-Ka Wong

**Affiliations:** a Cardiology Division, Department of Medicine, School of Clinical Medicine, Li Ka Shing Faculty of Medicine, The University of Hong Kong, Hong Kong SAR, China; b Cardiology Division, Department of Medicine, Queen Mary Hospital, Hong Kong SAR, China; c Cardiac Medical Unit, Grantham Hospital, Hong Kong SAR, China; d Department of Cardiothoracic Surgery, Queen Mary Hospital, Hong Kong SAR, China; e Department of Cardiovascular Surgery, Mount Sinai Health System, New York, New York, USA; f Cambridge Stem Cell Institute, The University of Cambridge, Cambridge, United Kingdom

**Keywords:** transcatheter edge-to-edge repair (TEER), tricuspid regurgitation (TR)

Tricuspid transcatheter edge-to-edge repair (T-TEER) is a promising therapy for tricuspid regurgitation (TR).^1^, ^2^, ^3^ T-TEER data in Asia with a dedicated system remain scarce.^4^ We report a retrospective single-center observational study of patients who underwent T-TEER with TriClip or MitraClip (Abbott) using the “miskey” technique in Hong Kong.

## Methods

The study was approved by the HKU/HA HKW Institutional Review Board (UW-12-177). Consecutive patients aged >18 years with severe TR treated with TriClip or MitraClip with the “miskey” technique at Queen Mary Hospital, Hong Kong, between January 2021 and February 2025 were included. The “miskey” technique refers to the insertion of a MitraClip delivery catheter with 90° counter-clockwise rotation to facilitate T-TEER.

All patients had TR severity classified into 5 grades.^5^ Vena contracta, regurgitant volume, and regurgitant jet area were measured. Tricuspid annulus and pressure gradients were measured. Right ventricular volume, fractional area change, tricuspid annular plane systolic excursion, right atrial volume, and inferior vena cava dimensions were evaluated.

Primary outcome was TR severity 30 days following successful T-TEER. Rate of successful implantation, device embolization, migration, and single leaflet device attachment (SLDA) were reported. Clinical events were defined according to Tricuspid Valve Academic Research Consortium (TVARC) criteria.^6^ Major adverse events (MAE) comprising TVARC clinical events were evaluated.

Normality was tested using the Shapiro-Wilk test. Comparisons were performed using Student's *t*-tests and the Wilcoxon test, chi-square and Fisher’s exact test, Wilcoxon signed-rank and Friedman tests, and Pearson’s correlation. *P* < 0.05 was considered significant.

## Results

### Baseline characteristics

A total of 78 patients (75.0 years [68.5-81.8]; 47.4% female) including 87.2% with secondary TR, were included. TR severity was classified as severe (3+) in 21.8%, massive (4+) in 55.1%, and torrential (5+) in 23.1%; 64.2% of patients were NYHA functional class III/IV. Mean body mass index was 21.4 ± 3.51 (weight 55.3 ± 10.8 kg; height 160 ± 8.70 cm). Top comorbidities included atrial fibrillation (87.2%), hypertension (60.3%), and chronic kidney disease (57.7%). Subsets of patients had interventions on mitral (21.8%), aortic (10.3%), and tricuspid valves (8.97%); 15.4% had a cardiac implantable electronic device. Median European System for Cardiac Operative Risk Evaluation-II was 5.50% (3.09-10.3).^7^ Median TRI-SCORE was 5.00 (2.25-6.00), with predicted in-hospital mortality of 14.0% (3.50%-22.0%).^8^ Cardiogenic shock was present in 9 (11.5%).

### T-TEER procedure

Procedures were performed under general anesthesia with transesophageal echocardiography. Three-dimensional intracardiac echocardiography was used in 22 (28.2%). Successful implantation was achieved in 76 patients (97.4%), including 66 with TriClip and 10 with MitraClip. Thirty-five (44.9%) required concomitant left-sided intervention.

Among 76 patients with successful implantation, 1 device was implanted in 47.4% (n = 36), 2 in 39.5% (n = 30), and 3 in 13.2% (n = 10). XTWs were implanted in all patients, with a median 1.50 (1.00-2.00) clips per patient. XTs were utilized in 3 patients (3.95%). Sites of implantation included antero-septal (97.4%), postero-septal (15.8%), and antero-posterior (1.32%). SLDA occurred in 3.85% of patients (3 of 78) and 2.38% of devices (3 of 126). There was no embolization or migration. Among patients with successful implantation, 90.8% were prescribed an anticoagulant, 7.89% an antiplatelet agent, and 1.32% no antithrombotic.

**Figure 1:**
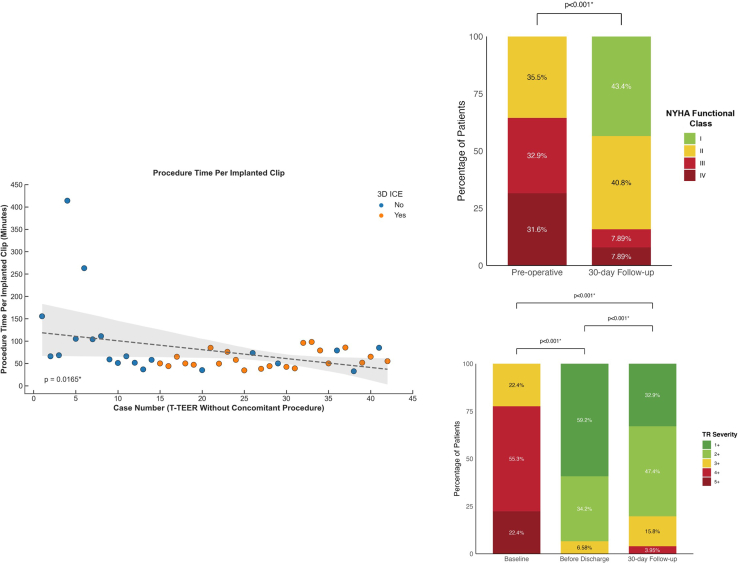
T-TEER in Asian Patients Successful implantation of tricuspid transcatheter edge-to-edge repair **(**T-TEER) devices was achieved in 76 patients (97.4%), including 66 with TriClip and 10 with MitraClip using the “miskey” technique. Among the 76 patients with successful T-TEER implantation, 66 patients (86.6%) showed improved NYHA functional class, with class III/IV decreasing from 64.5% to 15.8% at 30 days (*P* < 0.001∗). The proportion with ≥3+ tricuspid regurgitation (TR) reduced to 6.58% before discharge and 19.7% at 30 days (*P* < 0.001∗). Among 42 patients who underwent standalone T-TEER without concomitant left-sided procedure, a significant trend toward shorter procedure times per implanted clip (*P* = 0.0152∗) was observed over time. The median procedure time per clip decreased from 105 minutes (68.3-156) in the initial 5 cases to 55.0 minutes (51.7-65.0) in the most recent 5 cases (*P* = 0.0317∗). 3D ICE = 3-dimensional intracardiac echocardiography.

Forty-two patients who underwent standalone T-TEER revealed a trend toward shorter procedure time per clip (*P* = 0.0152∗) (Figure 1) with median of 105 minutes (68.3-156) in the first 5 cases and 55.0 minutes (51.7-65.0) in the last 5 (*P* = 0.0317∗). There was no significant difference in successful implantation rate in the first vs last 50% of cases (*P* = 0.494).

### Clinical outcomes

MAEs occurred in 4 patients (5.13%) in 30 days. Type 3 or above bleeding and vascular complications each occurred in 1 (1.28%). All-cause mortality occurred in 3 (3.85%), including 2 (2.56%) with cardiovascular mortality from left-sided heart failure. Acute kidney injury occurred in 1 (1.28%). There was no significant difference in the MAE rate when stratified by T-TEER vs T-TEER with concomitant left-sided intervention (*P* = 0.321) or first vs last 50% of cases (*P* = 0.615). After successful T-TEER, 66 (86.6%) showed improved NYHA functional class, with class III/IV decreasing from 64.5% to 15.8% at 30 days (*P* < 0.001∗) (Figure 1).

### Echocardiography

Among 76 patients with successful implantation, 5 (6.58%) patients had ≥3+TR before discharge (*P* < 0.001∗) (Figure 1). At 30 days, 15 (19.7%) had ≥3+TR, with no significant difference between first vs last 50% of cases (*P* = 0.386). Effective regurgitation orifice area reduced from 0.665 cm^2^ (0.530-0.812) to 0.250 cm^2^ (0.140-0.340) (*P* < 0.001∗). Vena contracta width reduced from 0.900 cm (0.800-1.28) to 0.400 cm (0.300-0.500) (*P* < 0.001∗). Regurgitant volume declined from 55.7 mL/beat (47.6-68.3) to 17.6 mL/beat (10.2-24.6) (*P* < 0.001∗). Regurgitant jet area decreased from 13.4 cm^2^ (10.2-19.4) to 5.39 cm^2^ (3.05-8.25) (*P* < 0.001∗). Peak and mean pressure gradient increased from 2.50 mm Hg (2.00-4.00) to 3.50 mm Hg (2.00-6.00) (*P* < 0.001∗), and 1.00 mm Hg (1.00-1.00) to 2.00 mm Hg (1.00-2.75) (*P* < 0.001∗), respectively. No significant changes in right ventricular dimension, right ventricular function, right atrial volume, or inferior vena cava diameter were observed. A subset of 72 patients had echocardiography repeated in 6 to 12 months and 16.7% patients had ≥3+TR. (∗ indicates *P* < 0.05).

## Discussion

We demonstrated high procedural success rate for T-TEER involving the TriClip system in an Asian population, with substantial TR reduction and functional improvement. Shorter procedure time with increasing experience was observed.

Among patients with successful implantation, only 6.58% had ≥3+TR before discharge. At 30 days, a higher proportion of patients had ≥3+TR (19.7%). This could be partly attributable to the difference in fluid management in hospital vs at home.^9^ At 6 to 12 months, only 16.7% patients had ≥3+TR, comparable with TRILUMINATE (Trial to Evaluate Cardiovascular Outcomes in Patients Treated With the Tricuspid Valve Repair System Pivotal) results.^3^

Our real-world patient cohort demonstrated high acuity, including 11.5% with cardiogenic shock and 44.9% with left-sided disease requiring intervention. At 30 days, 2 patients (2.56%) succumbed to left-sided heart failure, substantially lower than the TRI-SCORE predicted rate. SLDA occurred in 2.38% of implanted devices, similar to the bRIGHT (An Observational Real-World Study Evaluating Severe Tricuspid Regurgitation Patients Treated With the Abbott TriClip Device) study results (3.5%).^10^ As dedicated T-TEER systems become more widely available in Asia, larger-scale studies may be conducted to evaluate generalizability of our findings.

Small sample size may reduce the power to detect significant differences, such as MAE rates. Although the use of M-TEER systems with the “miskey” technique was common during the study, it remains relatively uncommon in regions where dedicated T-TEER systems are available. Finally, the 6-minute walk test and Kansas City Cardiomyopathy Questionnaire were not available.

## Conclusions

T-TEER is safe and effective for severe TR in Asian patients, with high procedural success rate, causing TR reduction, and functional improvement.

## Funding Support and Author Disclosures

Dr Tang has received honoraria for speaking from Medtronic; has served as a physician proctor, consultant, advisory board member, TAVR publications committee member, APOLLO trial screening committee member, and IMPACT MR steering committee member for Medtronic; has received honoraria for speaking from Abbott Structural Heart; has served as a physician proctor, consultant, advisory board member, and TRILUMINATE trial anatomic eligibility and publications committee member for Abbott Structural Heart; has served as an advisory board member for Boston Scientific and JenaValve; has served as a consultant and physician screening committee member for Shockwave Medical; has served as a consultant for Philips, Edwards Lifesciences, Anteris, Peija Medical, and Shenqi Medical Technology; and has received honoraria for speaking from Siemens Healthineers. All other authors have reported that they have no relationships relevant to the contents of this paper to disclose.
